# Enhanced Efficacy of Bleomycin in Bladder Cancer Cells by Photochemical Internalization

**DOI:** 10.1155/2014/921296

**Published:** 2014-06-30

**Authors:** Yan Baglo, Lars Hagen, Anders Høgset, Finn Drabløs, Marit Otterlei, Odrun A. Gederaas

**Affiliations:** ^1^Department of Cancer Research and Molecular Medicine, Faculty of Medicine, Norwegian University of Science and Technology, P.O. Box 8905, 7491 Trondheim, Norway; ^2^PCI Biotech AS, Strandveien 55, 1366 Lysaker, Norway; ^3^APIM Therapeutics AS, Sem Sælandsvei 14, 7084 Trondheim, Norway

## Abstract

Bleomycin is a cytotoxic chemotherapeutic agent widely used in cancer treatment. However, its efficacy in different cancers is low, possibly due to limited cellular internalization. In this study, a novel approach known as photochemical internalization (PCI) was explored to enhance bleomycin delivery in bladder cancer cells (human T24 and rat AY-27), as bladder cancer is a potential indication for use of PCI with bleomycin. The PCI technique was mediated by the amphiphilic photosensitizer disulfonated tetraphenyl chlorin (TPCS_2a_) and blue light (435 nm). Two additional strategies were explored to further enhance the cytotoxicity of bleomycin; a novel peptide drug ATX-101 which is known to impair DNA damage responses, and the protease inhibitor E-64 which may reduce bleomycin degradation by inhibition of bleomycin hydrolase. Our results demonstrate that the PCI technique enhances the bleomycin effect under appropriate conditions, and importantly we show that PCI-bleomycin treatment leads to increased levels of DNA damage supporting that the observed effect is due to increased bleomycin uptake. Impairing the DNA damage responses by ATX-101 further enhances the efficacy of the PCI-bleomycin treatment, while inhibiting the bleomycin hydrolase does not.

## 1. Introduction

Bladder cancer is one of the most common cancers in the world and causes more than 100 000 deaths every year [1, 2]. In Norway bladder cancer has been one of the five most common cancer types for men during the last ten years [3] and in United States it is estimated that 74 690 new cases and 15 580 deaths will be reported in 2014 [4]. Approximately 70–80% of diagnosed bladder cancers worldwide are nonmuscular invasive bladder cancer (NMIBC) for which intravesical chemotherapy is used as an adjuvant treatment to the standard transurethral resection [1]. However, significant improvements in preventing disease progression and recurrence have not been obtained [1, 2]. Due to high intrinsic cytotoxicity and low myelosuppression and immune-suppression, bleomycin is used in the treatment of cancers such as malignant lymphomas, testicular carcinomas, and squamous cell carcinomas (see review by Ramotar and Wang [5] and references therein). However, good clinic efficacy has not been found in bladder cancer [6–8]. This could be due to low uptake into the cells as bleomycin consists of rather large water-soluble glycopeptidic molecules that are most likely unable to cross cell membranes by passive diffusion, and, thus, rely on endocytosis and/or transporters [9, 10]. Studies have shown that cellular responses to bleomycin are cell type-dependent. In cell lines with low sensitivity to bleomycin, the drug resistance is considered mainly to be due to membrane barrier, degradation by hydrolases in lysosomes or bleomycin hydrolase (BLMH) in the cytosol, elevated DNA repair capacity, and low activity of bleomycin transporters [9–13]. Severe side effects of bleomycin at high dose are therefore limiting its clinical applications [5]. In an effort to overcome the membrane barrier for bleomycin, electropermeabilization was shown to enhance the efficacy [14, 15].

The PCI technology has been developed from photodynamic therapy (PDT) as an efficient drug delivery tool to enhance the effect of several types of therapeutic molecules [16]. In the PCI technology a membrane-embedded photosensitizer is used together with a therapeutic agent by endocytotic delivery [17, 18]. The photosensitizers used in PCI, such as mesotetraphenyl chlorin disulfonate (TPCS_2a_) used in this study, are designed as amphiphilic molecules that initially localize to the plasma membrane, but later are incorporated into the endosomal membranes by endocytosis [19–21]. When used together with bleomycin, the bleomycin molecules are enclosed in the endocytotic vesicles, and exposure to light leads to endosomal rupture by phototoxic damage and release of bleomycin molecules into the cytosol [22, 23]. Side effects often seen in the conventional systemic therapeutic strategies can also be reduced by PCI because the enhanced effect is localized to the area exposed to light [24, 25]. Enhanced efficacy of PCI with bleomycin has been documented in several preclinical studies and clinical trials [24–28]. A phase I clinical trial of TPCS_2a_-mediated PCI of bleomycin showed no severe side effects associated with the treatment, and the efficacy and safety of the modality are currently being evaluated in a phase II interventional clinical trial [27, 29].

Once inside the nucleus, bleomycin-induced DNA strand breaks are leading to apoptosis, extended cell cycle arrest, mitotic cell death and increased risk of chromosome aberrations if not properly repaired [30–32]. A novel designed cell-penetrating peptide named ATX-101, containing the AlkB homolog 2 PCNA-interacting motif (APIM), has been shown to enhance cytotoxicity of several chemotherapeutic drugs [33]. The APIM-motif mediates interaction with proliferating cell nuclear antigen (PCNA) in many proteins involved in DNA repair, apoptosis, and restart of replication and cell cycle regulation after DNA damage. Impairing the interactions between these proteins and PCNA by ATX-101 impairs the cellular DNA damage responses and thus sensitizes the cells to chemotherapeutic drugs [33–37]. In this study, cytotoxicity of TPCS_2a_-mediated PCI of bleomycin (PCI-bleomycin) was studied in rat bladder cancer cells (AY-27) and human bladder cancer cells (T24) using the PCI strategy of illumination after bleomycin treatment [38, 39]. For all experiments, a human epidermoid carcinoma cell line (A431) was used as reference due to its known sensitivity to PCI [40]. Furthermore, we examined the effects of inhibiting the DNA damage response and bleomycin degradation in combination with PCI-bleomycin. The levels of induced DNA damages were investigated using the comet assay. Our results demonstrate that PCI enhances bleomycin efficacy in human and rat bladder cancer cells under optimal conditions. Combination therapy using PCI-bleomycin and ATX-101 further enhances the observed cytotoxicity.

## 2. Materials and Methods

### 2.1. Cell Culture

Rat bladder transitional carcinoma cells (AY-27) were maintained using the same RPMI culture medium and conditions as described in our earlier study [41]. Human epidermoid carcinoma cells (A431) and human bladder carcinoma cells (T24) were maintained in Dulbecco's Modified Eagle's Medium (D6429) supplemented with 2 mM L-glutamine, 1% penicillin/streptomycin, 10 mM HEPES, 1 mM sodium pyruvate (Lonza), and 10% (v/v) fetal bovine serum. All medium chemicals were purchased from Sigma except noted.

### 2.2. Chemicals

TPCS_2a_ (30 mg mL^−1^, Amphinex) dissolved in Tween 80 and 50 mM Tris buffer was provided by PCI Biotech AS (Oslo, Norway) and stored at 4°C in aliquots. The stock was first diluted with 50 mM Tris phosphate buffer (pH 8.5) to 0.06 mg mL^−1^ and further diluted with fresh culture medium immediately before use. All work with TPCS_2a_ was performed under subdued light or light protection.

Bleomycin powder (15000 IU, Baxter, Norway) was dissolved with 0.9% salt water as stock (1000 IU mL^−1^) and stored at −20°C in aliquots. Peptide drug ATX-101 (1 mM) was supplied by APIM Therapeutics AS (Trondheim, Norway) and stored at 4°C in aliquots. Protease inhibitor E-64 powder (Sigma), a known inhibitor of bleomycin hydrolase [42, 43], was diluted with deionized water as stock (1 mM) and stored at −20°C in aliquots. Resazurin sodium salt powder (Sigma) was dissolved with PBS to 2.5 mM followed by filtering and sonication under subdued light. Resazurin stock was stored at −20°C in aliquots. These chemical stocks were further diluted to desired concentrations with fresh culture medium immediately before use.

DRAQ5 solution (5 mM, BioStatus Limited, UK), a novel DNA-detecting far-red-fluorescing dye [44], was stored at 4°C under light protection and diluted with PBS (1 : 10000) under subdued light immediately before use.

### 2.3. Light Source

Blue light with *λ*_max⁡_ of 435 nm used in this study was available from a LumiSource lamp (PCI Biotech AS, Norway). The lamp is designed specifically to provide stable and homogenous fluency with an irradiance of 12.9 mW cm^−2^ over a defined illumination area, allowing attached living cells to be illuminated from the bottom of culture dishes or plates. With the light doses used in the PCI experiments, the photosensitizer is not likely to be affected by photobleaching [45].

### 2.4. Cellular Uptake of TPCS_2a_

Cells were seeded out into 96-well plates (6000 cells/well, CytoOne, USA Scientific, Inc.). Attached cells were incubated with TPCS_2a_ at a series of concentrations (18 h). Subsequent to removal of TPCS_2a_-medium, fluorescence intensity of cellular accumulated TPCS_2a_ was measured using FLUOStar Omega microplate reader (410 nm/650 nm, BMG Labtech GmbH, Germany). The cells were immediately washed with cold PBS, fixed with fresh 2% paraformaldehyde (150 *μ*L/well, 15 min, on ice, no shaking), and stained with 0.5 *μ*M DRAQ5 (50 *μ*L/well, 20 min, RT, gentle shaking, in the dark). The plates were dried out after washing with cold PBS. Fluorescence intensity of DNA binding DRAQ5 was measured using Odyssey Imager at 700 nm channel according to the user manual (Li-Cor Infrared imaging system, LI-COR Biosciences, Ltd., UK). Cellular accumulation of TPCS_2a_ was determined by dividing relative fluorescence intensity (normalized with control cells) of TPCS_2a_ with fluorescence intensity of DRAQ5 (DNA content in the same well).

### 2.5. Resazurin Survival Assay

Control and treated cells were incubated with resazurin medium (200 *μ*M, 130 *μ*L/well, 2-3 h, 37°C) under light protection and fluorescence intensity was measured using the FLUOStar Omega microplate reader (544 nm/590 nm). Cell survival fraction was determined after normalization with fluorescence intensity of resazurin medium and control cells.

### 2.6. Cytotoxicity Assays

Cells were seeded out into 96-well plates (6000 cells/well). Attached cells were treated using one of the protocols below.

*Protocol A (photodynamic treatment)*. The cells were incubated with TPCS_2a_ for 18 h. Subsequent to removal of TPCS_2a_-medium, cells were washed with culture medium twice and chased (4 h) in fresh drug-free medium before exposure to blue light at different intervals [26]. 

*Protocol B (drug treatment)*. The cells were (co-)incubated with drug(s) (bleomycin, ATX-101 or E-64) for 4 h and then washed once with culture medium. 

*Protocol C (PCI-bleomycin treatment)*. Here protocol B was incorporated into protocol A after TPCS_2a_-treatment. The treated cells were (co-)incubated with bleomycin (and ATX-101/E-64) for 4 h instead of being chased in drug-free medium. The cells were washed once before illumination. Therapeutic drug and light doses are listed in Table 1.

Finally, cell survival fraction was determined by resazurin survival assay after postincubation for 48 h. The time point of 48 h was selected based on bleomycin effect measured at each day from 1 to 7 days after bleomycin treatment in these cell lines (see Figure 1S in Supplementary material available online at http://dx.doi.org/10.1155/2014/921296 and protocol D in Materials and Methods, Section 2.9). Cell survival fractions showed the same relative relationship among the three cell lines across the time series. Considering the subsequent studies on bleomycin effects in different combined treatments, cell growth measured at 48 h after bleomycin treatment seemed to be an optimal time point with potential to show clear differences in treatment effect in all cell lines that were used. Clonogenic assay was also performed in T24 and AY-27 cell lines as method control, showing that T24 had weaker colony forming capacity than AY-27. However, due to possibly misleading results caused by differences in colony forming capacity rather than drug effects, clonogenic assay was not used for the actual study (see Figure 2S in Supplementary Information and protocol E in Materials and Methods, Section 2.9).

When using protocol C, the cells were washed one additional time compared to protocol A. This could eliminate cellular TPCS_2a_ and thereby reduce the photodynamic cytotoxicity to a small extent in cells treated with PCI-drug(s) (see Figure 3S in Supplementary Information). However, this additional washing also favored the PCI-enhanced drug effect by reducing the effect of PDT in the experiment. Thus, the experiments with lowest PDT effect were selected as representative results.

### 2.7. Analysis of BLMH Expression by Western Blot

Protein lysate was extracted from (treated) cells using the same protocol as described in an earlier study [41]. Briefly, the cell pellet was resuspended in lysis buffer followed by sonication. Protein concentration was determined using Bio-Rad protein assay after centrifugation.

Expression of bleomycin hydrolase (BLMH) was analyzed by 1D Western blot using a standard protocol (Invitrogen). As previously described [34], protein lysates (100 *μ*g) were separated on 10% Bis-Tris gel (NuPAGE, Invitrogen) and transferred onto PVDF membrane (Immobilon, Millipore). Proteins were further detected using anti-BLMH primary antibody (ab77111, Abcam), HRP-conjugated rabbit anti-mouse secondary antibody (p0260, Dako Denamark), and anti-PCNA antibody (ab29, Abcam) as loading control. K562 whole cell lysate (30 *μ*g, ab7911, Abcam) was used as positive control of the primary antibody.

### 2.8. Comet Assay

Cells were seeded out into 6-well plates (5 × 10^4^ cells/well, CytoOne). Bleomycin or PCI-bleomycin treated cells were washed with PBS and then detached with accutase (15 min, 37°C, Sigma) immediately after treatment or after an interval of postincubation (15 min and 30 min, resp.) in culture medium. DNA damage level was further evaluated using a standard protocol of single cell gel electrophoresis [46–48]. As previously described [48], the cells were resuspended at 37°C in 1% low-melt agar after centrifugation and loaded onto a precoated microscope slide which was immediately cooled on ice. The embedded cells were lysed in fresh cold lysis buffer [48] overnight and then electrophoresed in cold alkaline buffer (pH 13.3), thus the comet assay detects single and double strand DNA breaks, abasic sites, and repair intermediates. DNA fragments stained with ethidium bromide were visualized in an inverted fluorescence microscope (Zeiss Axiovert 200M, Germany) equipped with a Sony XCD-X700 camera. Several hundreds of comets in each sample were evaluated using Komet 5.5 imaging software (Andor Technology).

### 2.9. Cytotoxicity Assays Used in the Supplementary Information

*Protocol D (long-term bleomycin cytotoxicity)*. Cells were seeded out in 25 cm^2^ culture flasks (1 × 10^6^ cells/flask) and treated with bleomycin (4 h) at desired concentrations. The cells were washed with PBS and detached with trypsin before reseeding into 96-well plates (1000 cells/well). Cell survival fraction was determined by resazurin survival assay from the next day, day 1 until day 7.

*Protocol E (clonogenic assay)*. Cells were seeded out and treated with bleomycin (4 h) as described in Protocol D before reseeding into petri dishes (100 cells/dish). The cells were incubated with culture medium for 7–10 days allowing colony formation. After washing with PBS, the colonies were fixed with 6% glutaraldehyde (30 min) and then stained with 0.5% crystal violet (30 min). Plating efficiency (PE) and surviving fraction (SF) were calculated after counting the air-dried colonies (PE = number of colonies counted/number of cells plated × 100%; SF = PE of treated sample/PE of control × 100%).

## 3. Results and Discussion

### 3.1. Cellular Uptake of TPCS_2a_ and Dose Responses to Blue Light and Bleomycin

**Figure 1:**
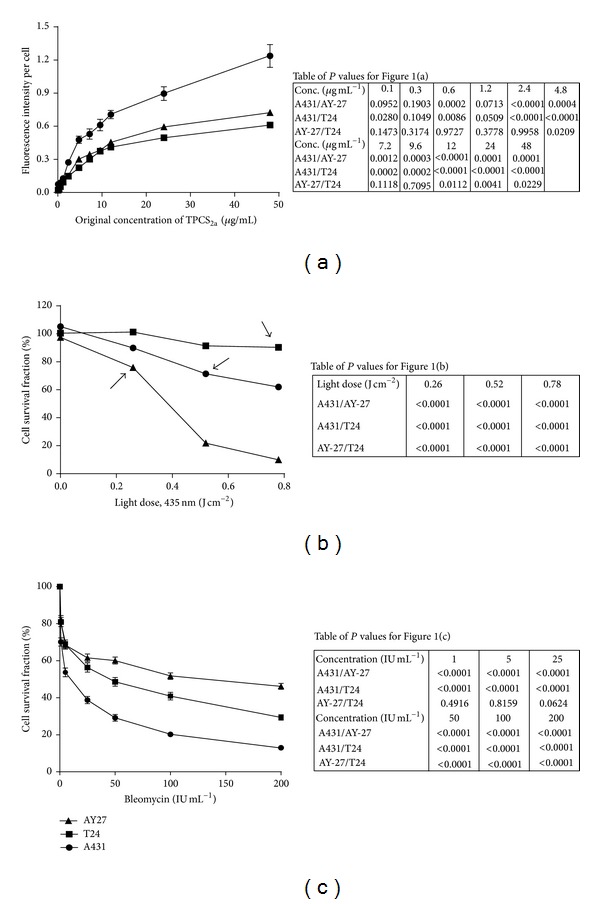
Cellular uptake of TPCS_2a_ is dose-dependent, its cytotoxicity is dependent upon dose of blue light, and bleomycin cytotoxicity is cell type-dependent. (a) Cellular accumulation at increasing concentrations of TPCS_2a_ in A431, T24, and AY-27 cells. After 18 h the TPCS_2a_ fluorescence was normalized to fluorescence intensity per cell by DRAQ5-staining. The data are from one representative experiment out of two (mean of 12 wells ± SEM). (b) Cytotoxicity of increasing blue light doses (435 nm) in A431, T24, and AY-27 cells exposed to 0.3 *μ*g mL^−1^  TPCS_2a_ for 18 h. The data are from one representative experiment out of three (mean of 24 wells ± SEM). Light doses selected for further PCI experiments are indicated with arrows. (c) Dose response to bleomycin determined as cell growth after 48 h. A431, T24, and AY-27 cells were treated with different bleomycin doses for 4 h. The data are from one representative experiment out of four (mean from 48 wells ± SEM). Tables of *P* values show statistical significance using two-tailed Student's *t*-test.

The uptake of TPCS_2a_ to the endosomal membrane is essential for drug delivery. To assess the uptake capacity in the bladder cancer cell lines T24 and AY-27, compared to the reference skin cancer cell line A431 (earlier shown to have good PCI efficacy [40]), fluorescence intensity of cellular TPCS_2a_ was measured and normalized against cell count. The results showed that the uptake was dose-dependent in all three cell lines (Figure 1(a)). Importantly, a much higher uptake was seen in the skin cancer cell line A431, compared to the bladder cancer cell lines T24 and AY-27 (Figure 1(a)). The endocytotic rate is cell type-dependent [49], and this may account for differences in cellular TPCS_2a_ uptake as seen in these cell lines.

According to the principle of PCI, the applied doses of both photosensitizer and light (termed photodynamic dose in this paper) are supposed to be sublethal, leading mainly to damage to the endosomal/lysosomal membrane. Therefore we set out to determine the sublethal photodynamic doses before evaluating PCI-bleomycin efficacy. No dark toxicity (Figure 4S in Supplementary Information), TPCS_2a_ buffer toxicity (Tween 80 and 50 mM Tris buffer), or light toxicity was observed in any of the cell lines under the experimental conditions (data not shown), thus the cytotoxicity is dependent upon light activation of the TPCS_2a_ molecules. The results showed that T24 cells were resistant to activation by blue light although they had similar TPCS_2a_ uptake as the AY-27 cells that were very light sensitive (Figure 1(b)). A431 cells, which accumulated more TPCS_2a_ molecules than the two other cell lines, were medium sensitive to light activation (Figure 1(b)). Light doses that gave low (0–20%) reduction in cell survival in this experiment (Figure 1(b), arrows) were selected for the PCI-bleomycin experiments, but in the following experiments a further three-time reduction of the TPCS_2a_ concentration was applied in order to further reduce the background cytotoxicity. In summary, these results show that the cell lines accumulate different levels of TPCS_2a_, but that this did not directly correlate with their light sensitivity.

Next, we tested the bleomycin sensitivity of the different cell lines. We treated the cells with bleomycin for 4 hours and measured the cell growth after 48 hours. The results showed that the reference cell line A431 was the most and AY-27 the least sensitive cell line to bleomycin (Figure 1(c)).

### 3.2. The Effect of Bleomycin Is Increased in Combination with PCI

**Figure 2:**
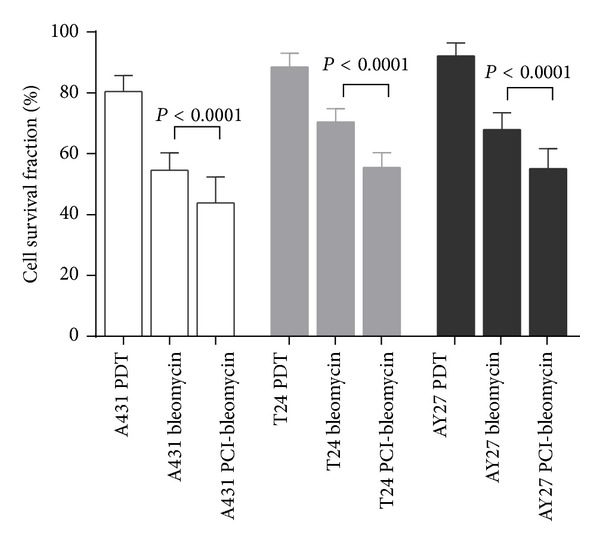
PCI enhances cytotoxic effect of bleomycin in all three cell lines. Cell survival fractions were determined 48 h after incubation with 0.1 *μ*g mL^−1^  TPCS_2a_ for 18 h, 50 IU mL^−1^ bleomycin or fresh medium for 4 h, and illumination (light doses as indicated in Figure 1(b)) in sequence. The data are from one representative experiment out of three (mean of 24 wells ± SD). *P* values were calculated using two-tailed Student's *t*-test and the values (*P* < 0.0001) indicated significant differences between bleomycin and PCI-bleomycin treatments.

To determine the efficacy of PCI-bleomycin in the three cell lines, sublethal doses of light (see arrows in Figure 1(b)) and TPCS_2a_ (0.1 *μ*g mL^−1^) were used. *P* values were calculated using two-tailed Student's *t*-test and the values (*P* < 0.0001) indicated that differences in cytotoxicity between bleomycin versus PCI-bleomycin treatment were highly significant. In addition, the PDT effect included in PCI-bleomycin treatment in each cell line was less than its PDT control due to an additional washing (see Section 2.6 in Materials and Methods). The results showed that bleomycin cytotoxicity was enhanced up to 20% by PCI, independent of cell type (Figure 2). This is likely due to increased uptake of bleomycin in the cells. At the selected conditions, A431 cells were more sensitive than the bladder cancer cells (44% surviving cells versus 55%, respectively, Figure 2). This probably reflects that this reference cell line had highest uptake of TPCS_2a_ in addition to being the most sensitive towards bleomycin (Figures 1(a) and 1(c)). These results are in agreement with recent results reported by Arentsen et al. [28]. Since PCI-bleomycin effect was still shown to be lower in the bladder cancer cell lines than in the reference skin cell line, two additional combination strategies to improve the effect were explored as shown in the next two sections.

### 3.3. Inhibition of Bleomycin Hydrolase Did Not Increase the Cytotoxicity of PCI-Bleomycin Treatment

**Figure 3:**
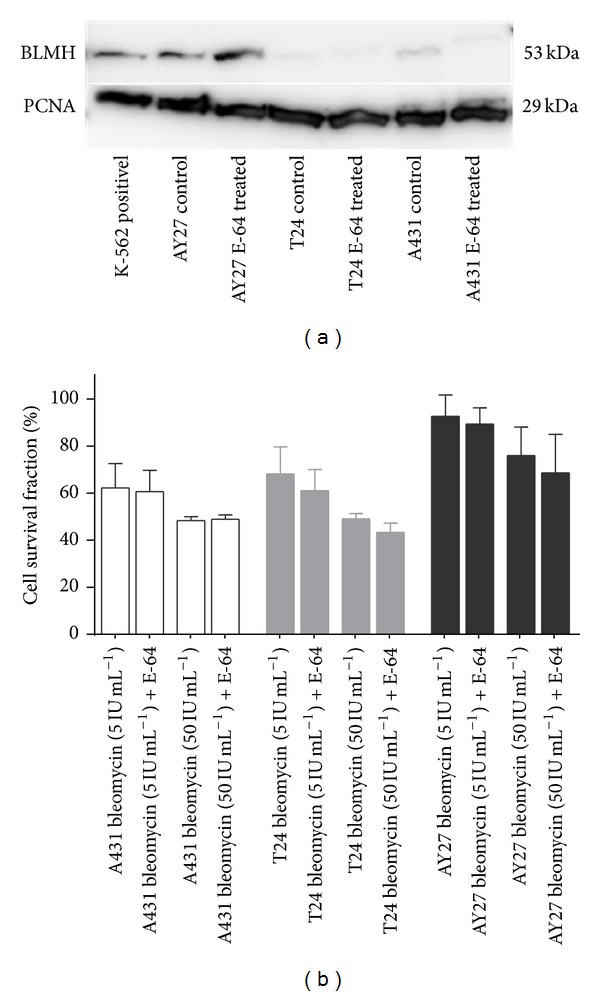
Bleomycin hydrolase is strongly expressed in AY-27 cells but the protease inhibitor E-64 does not sensitize AY-27 to bleomycin. (a) Western blots of BLMH expression in K562 (positive control), A431, T24, and AY-27 cells. E-64 treated cells are included as control. PCNA expression level is used as loading control. The image is from one representative experiment out of three. (b) Bleomycin cytotoxicity at two concentrations (5 and 50 IU mL^−1^) with and without E-64 treatment (concentrations used are listed in Table 1). Cell survival fractions were determined 48 h after 4 h-treatment with the drug(s). The data are from one representative experiment out of three (mean of 24 wells ± SD).

Different sensitivity towards bleomycin could be due to differences in uptake of the drug or differences in how the cells process the drug or repair the DNA damages induced by the drug. Bleomycin hydrolase (BLMH) is a cytosolic enzyme which has been shown to inactivate bleomycin before it enters the nucleus, and is, thus, believed to contribute to bleomycin resistance [11, 12, 30]. BLMH expression in the three cell lines was analyzed by Western blot (Figure 3(a)). The AY-27 cell line has 2-3 fold higher expression of BLMH than the other two cell lines. The BLMH inhibitor E-64 did not change the observed BLMH levels. AY-27 was the most bleomycin-resistant cell line (Figure 1(c)), also in presence of E-64 (Figure 3(b)), suggesting that BLMH is not important for the resistance of AY-27 towards bleomycin. The changes in sensitivity towards bleomycin in the other cell lines were also minimal following addition of E-64 (Figure 3(b)), indicating that BLMH is most likely not important for bleomycin sensitivity of these cells.

### 3.4. Impairing DNA Damage Responses during PCI-Bleomycin Treatment Increased the Efficacy

**Figure 4:**
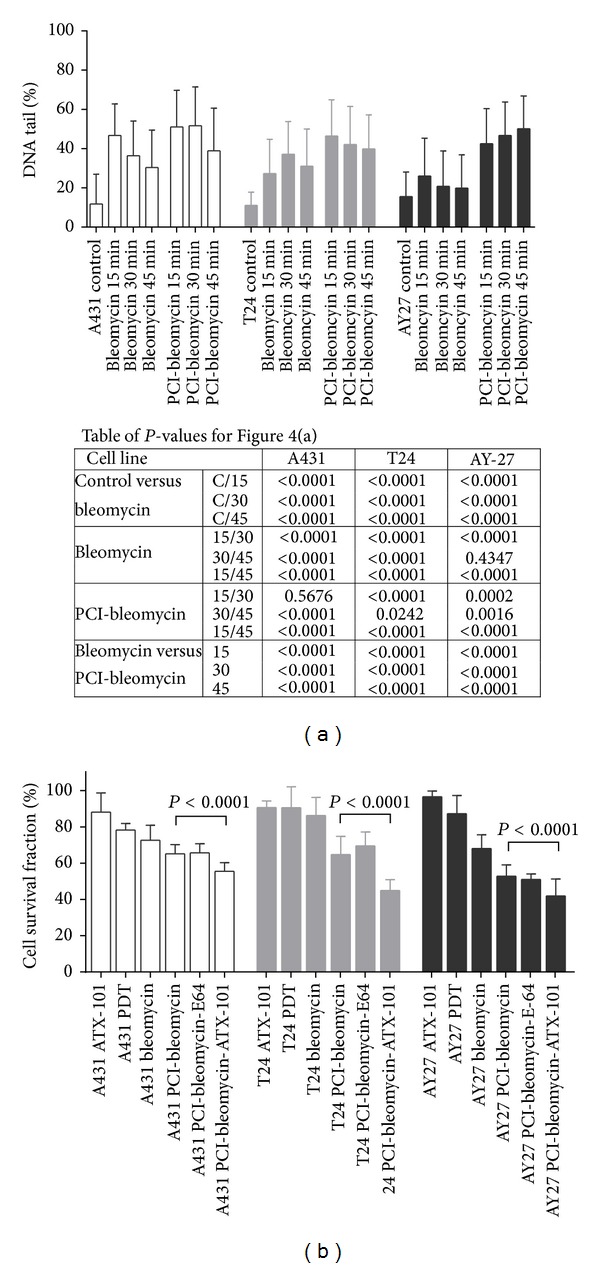
PCI elevates bleomycin-induced DNA damages and ATX-101 enhances PCI-bleomycin cytotoxicity. (a) Bleomycin and PCI-bleomycin induced DNA damages. Comet assay was performed at different intervals after incubation with 0.1 *μ*g mL^−1^  TPCS_2a_ (18 h), 50 IU mL^−1^ bleomycin (4 h), and illumination (light doses as indicated in Figure 1(b)) in sequence, or with the bleomycin treatment only. The data are from one representative experiment out of two experiments (mean of 500/700 cells ± SD). *P* values were calculated using two-tailed Student's *t*-test and the values indicated that the level of DNA damage between bleomycin treatment and PCI-bleomycin, or between different time intervals of the same treatment, was significantly different. (b) Cell growth after treatment with PDT, bleomycin, ATX-101, PCI-bleomycin, and PCI-bleomycin in combination with E-64 or ATX-101. Cell survival fractions were determined 48 h after incubation with TPCS_2a_ (18 h), bleomycin with/without ATX-101/E-64 (4 h), and light exposure in sequence (drug and light doses are shown in Table 1). Any PDT effect in the PCI combination treatments was reduced due to one additional washing (see Section 2.6 in Materials and Methods). The data are from one representative experiment out of three (mean of 12/24 wells ± SD). *P* values were calculated using Student's *t*-test and the values indicated that cell growth after bleomycin-bleomycin and PCI-bleomycin-ATX-101 treatment were significantly different.

Bleomycin binds to DNA strands resulting in both single stranded and double stranded DNA breaks. We measured the levels of DNA damage induced by bleomycin alone and by PCI-bleomycin using the comet assay. An increase in DNA damage by PCI-bleomycin versus only bleomycin would strongly indicate increased uptake of bleomycin in the cells. We also measured the DNA repair rate in each cell line. Because it takes 15 min to detach cells, the earliest time points for DNA damage evaluation were after 15 min. Cells were left to repair for additional 15 and 30 min (30 and 45 min in total, resp.) (Figure 4(a)). Statistical analysis was performed using two-tailed Student's *t*-test. Highly significant differences in DNA damage levels between treatments (*P* < 0.0001) are shown in Figure 4. Bleomycin-induced DNA damage was observed in all cell lines, but the levels at different time points varied between the cell lines. Bleomycin alone induced more DNA damage and the damage was removed more slowly in T24 and A431 than in AY-27 cells, consistent with the observation that AY-27 was more resistant to bleomycin. Furthermore, the results showed that PCI-bleomycin induced a higher level of DNA damage than bleomycin alone in all three cell lines (Figure 4(a)), supporting increased import of bleomycin. The highest increase in levels of DNA damage was seen in AY-27 cells, and importantly no reduction in the level of DNA damage could be detected 45 minutes after PCI-bleomycin treatment in this cell line, suggesting that the repair capacity was saturated. The observation that cell survival after 48 hours was 55% (see Figure 2) shows that a large fraction of the cells were eventually repaired, but at later time points.

The novel peptide drug ATX-101 has the potential to reduce several aspects of the cellular defense systems, including DNA repair, and is therefore enhancing the efficacy of several chemotherapeutics [33–35]. We tested if ATX-101 could increase the efficacy of PCI-bleomycin and found that ATX-101 enhanced the PCI-bleomycin efficacy with 14.7%, 30.5%, and 20.7% in A431, T24, and AY-27 cells, respectively, showing statistically significant differences (Figure 4(b)). The cytotoxic effects of the double combinations ATX-101-bleomycin and ATX-101-TPCS_2a_-PDT are similar or lower than PCI-bleomycin and lower than the triple combination ATX-101-PCI-bleomycin (data not shown). It should be taken into account that any PDT effect in these PCI combination treatments was reduced due to one additional washing (see Section 2). The additive effects of ATX-101 were stronger in the bladder cancer cells than in the skin cancer cells (reference cell line) under these experimental conditions (Figure 4(b)). Because the bladder cancer cells were less sensitive towards bleomycin both with regard to cell survival and induction of DNA damage (Figures 1 and 4) these results suggest that the cellular DNA repair capacity is a crucial factor for the efficacy of bleomycin, and therefore also the efficacy of PCI-bleomycin. As expected from the results in Figure 3(b), addition of E-64 did not affect PCI-bleomycin efficacy.

## 4. Conclusions

This is a fundamental study of specific aspects of the PCI technique. Using sublethal photodynamic doses, our results show that the cytotoxic effect of bleomycin is enhanced by TPCS_2a_-mediated PCI in the tested bladder cancer cells, although these cell lines show clear differences with respect to sensitivity to photosensitizer uptake, light dose, and DNA repair capacity. We show that the PCI technique elevates bleomycin-induced DNA damage levels in all three cell lines, strongly suggesting that more bleomycin molecules enter the nuclei compared to treatment with bleomycin as a single agent. Thus, the membrane barrier to bleomycin seems to be bypassed by the PCI technique. We have further demonstrated that application of a cocktail of PCI-bleomycin and ATX-101, under condition where individual drug levels have no or low toxicity, reaches a promising therapeutic effect in the human bladder cancer cells T24 which is two-fold stronger than in the reference cell line, the human skin cancer cells A431. Combining PCI-chemotherapy treatments with inhibitors of DNA repair therefore seems to be a promising therapeutic strategy for increased efficacy “on site.”

## Supplementary Material

Figure 1S. The long-term bleomycin cytotoxicity in the three cell lines AY-27, T24 and A431.
Figure 2S. Cell survival after bleomycin treatment in T24 and AY-27 cell line.
Figure 3S. Elimination of TPCS2a in AY-27 cells.
Figure 4S. No dark toxicity was observed in AY-27, A431 and T24 cells.
Figure 5S. Images of comet assay of AY-27, T24 and A431 cells.

## Figures and Tables

**Table 1:** Therapeutic drug and light doses (used in Figures 3(b) and 4(b)).

Cell line	TPCS_2a_	Light	Bleomycin	ATX-101	E-64
	(*μ*g mL^−1^)	(J cm^−2^)	(IU mL^−1^)	(*μ*M)	(*μ*M)
A431	0.2	0.387	5	5	10
T24	0.2	0.774	5	5	10
AY-27	0.1	0.387	50	5	50
