# Supplementary material for: Enhanced Efficacy of Bleomycin in Bladder Cancer Cells by Photochemical Internalization
Source: Biomed Res Int. 2014 Jun 30;2014:921296. doi: 10.1155/2014/921296 (PMC4101207; doi:10.1155/2014/921296)
Supplement: Supplementary file 1 — Figure 1S. The long-term bleomycin cytotoxicity in the three cell lines AY-27, T24 and A431. Figure 2S. Cell survival after bleomycin treatment in T24 and AY-27 cell line. Figure 3S. Elimination of TPCS2a in AY-27 cells. Figure 4S. No dark toxicity was observed in AY-27, A431 and T24 cells. Figure 5S. Images of comet assay of AY-27, T24 and A431 cells. [file 921296.f1.docx]

**Supplementary Information**

**Enhanced efficacy of bleomycin in bladder cancer cells by photochemical internalization**

Yan Baglo^1^, Lars Hagen^1^, Anders Høgset^2^, Finn Drabløs^1^, Marit Otterlei^1,3^ and Odrun A. Gederaas^1^

1. *Department of Cancer Research and Molecular Medicine, Faculty of Medicine, Norwegian University of Science and Technology, P.O.Box 8905, N-7491 Trondheim, Norway.*
2. *PCI Biotech AS, Strandveien 55, N-1366 Lysaker, Norway*
3. *APIM Therapeutics AS, Sem Sælandsvei 14, 7084 Trondheim, Norway*

Correspondence should be addressed to Yan Baglo; [yan.baglo@ntnu.no](mailto:yan.baglo@ntnu.no)

The results shown in Figure 1S, 2S and 3S are supplementary to the figures in the main paper. These results were used to determine an optimal time point (48 h) and support for the cytotoxicity assays (section 2.6 in Materials and methods of the main paper).

**Figure 1S (Supplementary).** The long-term bleomycin cytotoxicity in the three cell lines AY-27, T24 and A431. Cells were seeded out into 25 cm^2^ culture flasks (1×10^6^ cells/flask) and treated with bleomycin (4 h) at indicated concentrations (5, 50, and 100 IU ml^-1^) after cell attachment. The cells were re-plated into 96-well plates (1000 cells/well) with parallels. Cell survival fraction was determined by resazurin survival assay from the next day until the seventh. The data are from one representative experiments out of two (mean of 12/24 wells ± SD).

**Figure 2S (Supplementary).** Cell survival after bleomycin treatment in T24 and AY-27 cell line. Cells were seeded out into 25 cm^2^ culture flasks (1×10^6^ cells/flask) and treated with bleomycin (4 h) at indicated concentrations (5, 50, and 200 IU ml^-1^) after cell attachment. The cells were re-plated into petri dishes (100 cells/dish) with parallels. The fraction was determined and calibrated with the amount of colonies using a standard clonogenic assay. The time for colony forming was seven days for AY-27 cells and ten days for T24 cells.


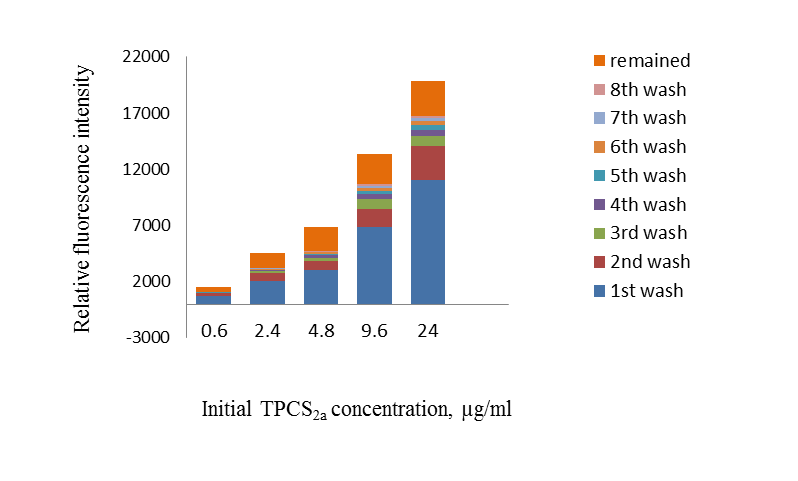


**Figure 3S (Supplementary)**. Elimination of TPCS_2a_ in AY-27 cells. Attached cells in 96-well plate were incubated with TPCS_2a_ (18 h) at different concentrations and washed with culture medium (100 µl/well) at every chase of 10-15 min. Chased medium was moved to a new 96-well plate in the same well position. The amount of released TPCS_2a_ and cellular retention after 8^th^ washing were determined by fluorescence measurement using FLUOStar Omega microplate reader (410 nm/640 nm, ±10 nm).

**Figure 4S (Supplementary)**. No dark toxicity was observed in AY-27, A431 and T24 cells exposed to 0.1, 0.2 and 0.3 µg ml^-1^ TPCS_2a_ for 18 h, respectively. The experiment was performed using Protocol B (manuscript) without exposure to blue light. The data are from one representative experiment out of three (mean of 24 wells ± SD). Among these, the data of 0.3 µg ml^-1^ TPCS_2a_ are from the same experiment shown in Figure 1B in the paper.


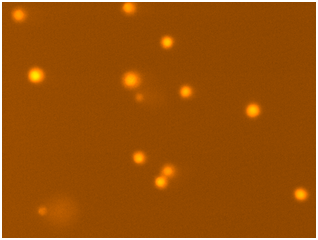


15min BLM


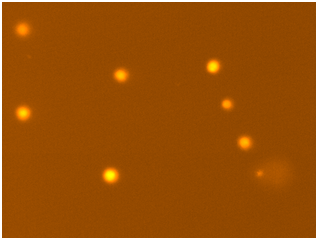


Control AY-27

AY-27

45min BLM

30min BLM


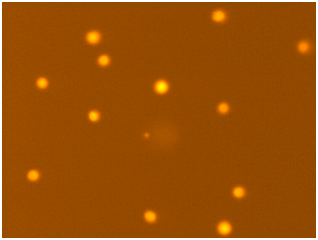

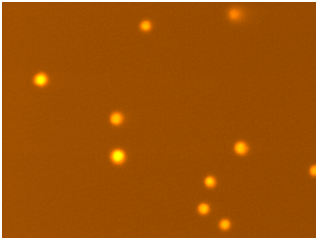


15min PCI-BLM


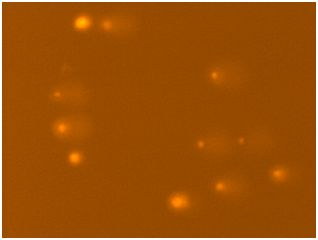


45min PCI-BLM

30min PCI-BLM


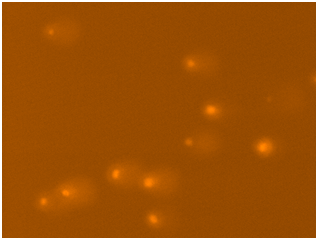

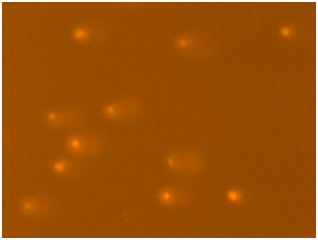

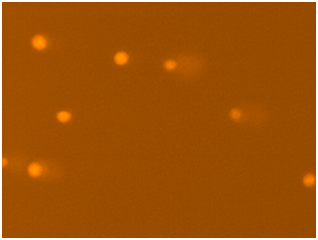


15min BLM

Control T24

T24

45min BLM

30min BLM

15min PCI-BLM

45min PCI-BLM

30min PCI-BLM


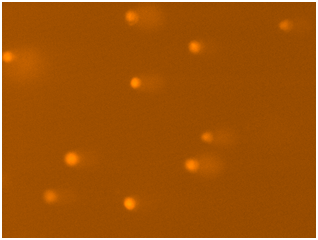

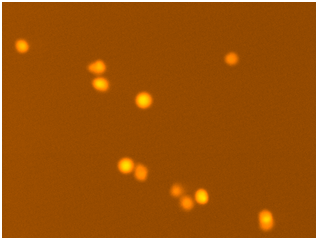

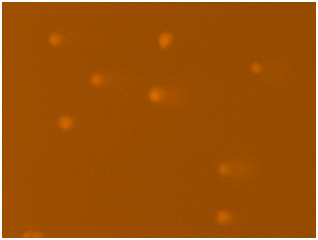

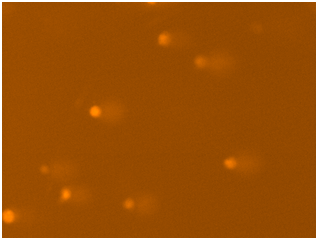

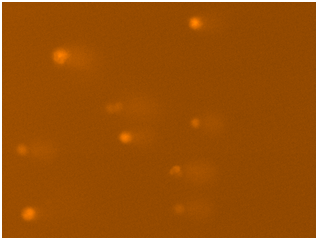

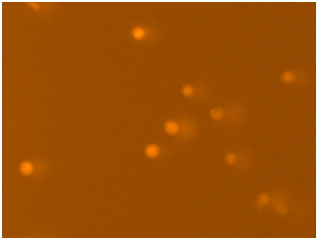

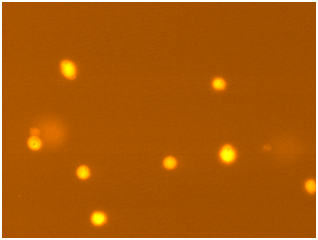


15min BLM

Control A431

A431

45min BLM

30min BLM

15min PCI-BLM

45min PCI-BLM

30min PCI-BLM


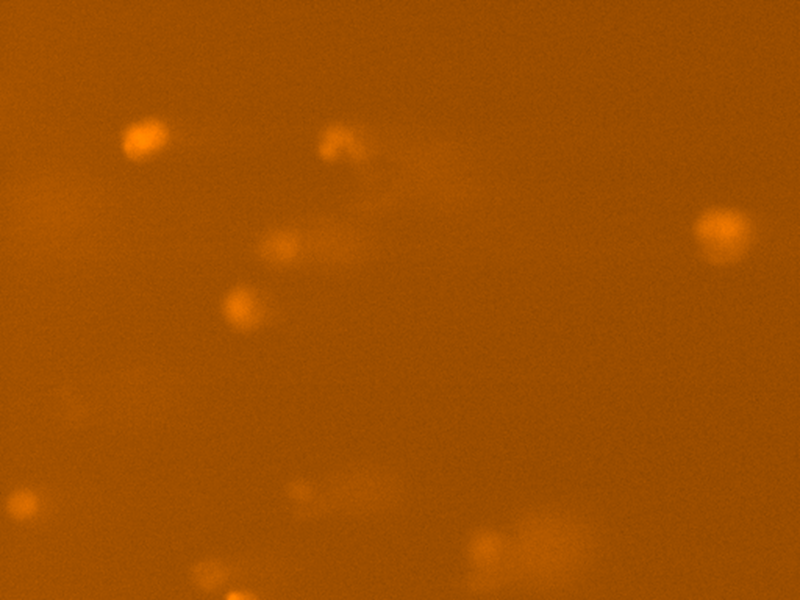

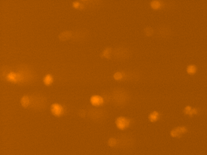

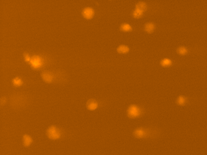

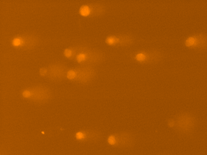

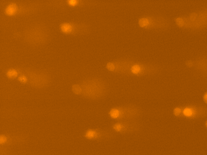

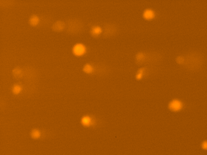


**Figure 5S (Supplementary).** Images of comet assay of AY-27, T24 and A431 cells. The experiment was performed and the images were taken as described in section 2.8 in Materials and methods. The data are shown in Figure 4A in the paper.
